# Study of a Synthetic Human Olfactory Receptor 17-4: Expression and Purification from an Inducible Mammalian Cell Line

**DOI:** 10.1371/journal.pone.0002920

**Published:** 2008-08-06

**Authors:** Brian L. Cook, Karin E. Ernberg, Hyeyoun Chung, Shuguang Zhang

**Affiliations:** Department of Biological Engineering and Center for Biomedical Engineering NE47-379, Massachusetts Institute of Technology, Cambridge, Massachusetts, United States of America; University of Arkansas for Medical Sciences, United States of America

## Abstract

In order to begin to study the structural and functional mechanisms of olfactory receptors, methods for milligram-scale purification are required. Here we demonstrate the production and expression of a synthetically engineered human olfactory receptor hOR17-4 gene in a stable tetracycline-inducible mammalian cell line (HEK293S). The olfactory receptor gene was fabricated from scratch using PCR-based gene-assembly, which facilitated codon optimization and attachment of a 9-residue bovine rhodopsin affinity tag for detection and purification. Induction of adherent cultures with tetracycline together with sodium butyrate led to hOR17-4 expression levels of ∼30 µg per 150 mm tissue culture plate. Fos-choline-based detergents proved highly capable of extracting the receptors, and fos-choline-14 (N-tetradecylphosphocholine) was selected for optimal solubilization and subsequent purification. Analysis by SDS-PAGE revealed both monomeric and dimeric receptor forms, as well as higher MW oligomeric species. A two-step purification method of immunoaffinity and size exclusion chromatography was optimized which enabled 0.13 milligrams of hOR17-4 monomer to be obtained at >90% purity. This high purity of hOR17-4 is not only suitable for secondary structural and functional analyses but also for subsequent crystallization trials. Thus, this system demonstrates the feasibility of purifying milligram quantities of the GPCR membrane protein hOR17-4 for fabrication of olfactory receptor-based bionic sensing device.

## Introduction

Membrane proteins are of vital importance to life, as evidenced by the fact that ∼30% of the genes in almost all sequenced genomes code for membrane proteins [1]–[3]. However, our understanding of the structures and functions of membrane proteins has lagged behind the known soluble proteins. As of June 2008, there are only 160 unique membrane protein structures known [http://blanco.biomol.uci.edu/Membrane_Proteins_xtal.html], which constitutes less than 1% of all known protein structures. The major bottleneck in obtaining membrane protein structures is the notorious difficulty involved in expressing and purifying the large quantities of membrane protein sample required for X-ray crystallography. In order to accelerate membrane protein structural and function studies, simple and reliable methods for membrane protein production must be developed.

Olfactory receptors (or odorant receptors) are an extremely large class of G-Protein Coupled Receptors (GPCRs) that function together combinatorially to allow discrimination between a wide range of volatile molecules [4], [5]. All GPCRs are integral membrane proteins with seven transmembrane domains arranged in a barrel-like conformation. In olfactory receptors, it is believed that this configuration forms a funnel-shaped pocket for odorant recognition [6]. The olfactory receptor (OR) gene family constitutes the largest single class of genes in the vertebrate genome (2–3% in the human). Current estimates put the number of human olfactory receptor genes at 636, with only 339 being functional and the rest being non-functional pseudogenes [7]. This is considerably less than the mouse OR gene family of 1209 (913 functional) [8] or the canine OR gene family of roughly 1200 (∼1000 functional) [9]. Despite the fact that they represent the largest class of known membrane proteins, no detailed structure exists for any olfactory receptor and the functional mechanisms of these amazing receptors remains unknown. The crucial first step to enable such pivotal studies is to engineer systems with the capacity to generate and purify milligram quantities of an olfactory receptor.

Mammalian olfactory receptors are expressed on the cilia of olfactory neurons within the nasal cavity. Odorant binding and recognition leads to activation and release of the olfactory G-protein G_olf_, which triggers cyclic-AMP production, ion-channel-mediated Ca2+ influx, and finally the firing of an action potential into the olfactory bulb to be interpreted by the brain [10]. Through an unknown mechanism of allelic inactivation, every olfactory neuron chooses a single OR gene to express. Signals from neurons that express the same olfactory receptor later converge downstream at neural foci called glomeruli [11]. As the same odorant will stimulate multiple ORs (and to various strengths), the brain receives a spatial map of receptor activity through these glomeruli [12]. Odorants are thought to be recognized by matching a specific spatial pattern (a combinatorial code) [5].

The human olfactory receptor 17-4 (hOR17-4, alternately known as OR1D2) is of particular interest since, in addition to olfactory neurons, it is also expressed on the midpiece of human spermatozoa [13]. Sperm expressing hOR17-4 were found to migrate towards known hOR17-4-responsive odorants such as bourgeonal. Thus the receptor serves a dual role in that it recognizes odorants in the nose as well as plays a potential role in sperm chemotaxis and fertilization. As structural studies of hOR17-4 would not only provide information crucial to understanding the molecular mechanism(s) of olfaction but also have application to human reproduction and population control, we thus selected this receptor as our prototype OR for expression and purification trials.

The GPCR family represents one of the most important known receptor classes as evidenced by the fact that half of all pharmaceutical drugs target GPCRs [14]. Despite their crucial role in mediating such diverse physiological processes as sight, smell, and the response to hormones and neurotransmitters, extremely little is known about these receptors at the structural level. A major breakthrough in 2007 was the determination of only the second GPCR crystal structure–that of a highly engineered human beta2-adrenergic receptor expressed in Sf9 insect cells where intracellular loop 3 was replaced with either antibody or T4 lysozyme to facilitate crystallization [15], [16]. Rhodopsin is perhaps the only GPCR that can be easily extracted from tissue, and this may explain why it was the first GPCR to have a detailed structure determined [17]–[19]. However, recent advances in the Khorana laboratory have led to the development of specific mammalian HEK293 cell lines for heterologous expression as well as methods for purification that yield milligram quantities of functional rhodopsin which is suitable for functional analysis and structural study [20]–[22]. We recently used same rho1D4-tag system for a one-step affinity purification of the human tetraspanin membrane protein CD81 [23]. Here we show that this system can be also adapted to facilitate the production and purification of another GPCR, the human olfactory receptor 17-4.

In order to carry out biochemical and structural analyses of olfactory receptors as well as engineer olfactory receptor-based biosensor devices, large quantities of receptors are required. Here we here report inducible expression, large-scale production of human olfactory receptor hOR17-4. We bioengineered the synthetic hOR17-4 gene into 50 oligonucleotide fragments, self-assembled them through high cycle PCR and inserted the assembled gene into an inducible human embryonic kidney cell line (HEK293S). We then induced its production using a combination of tetracycline and sodium butyrate. After systematic detergent screening, the zwitterionic detergent fos-choline-14 (FC14) was found to be most effective for solubilization and was subsequently used throughout the entire solubilization and purification. To our knowledge this is the first time an olfactory receptor has been purified from a mammalian cell line.

## Results

### Construction of synthetic hOR17-4-rho gene

To fabricate synthetic gene constructs we utilized a PCR-based method of gene synthesis [24] that involves parsing the DNA sequence into a set of small overlapping oligonucleotides. During an initial assembly PCR, these oligos function as both primer and template, while the DNA polymerase successively builds longer and longer fragments with each round of PCR. A second amplification PCR then enriches for the full-length gene (Figure S1). This process has more recently become known as PCA, or polymerase construction and amplification. To assist in parsing the sequence into oligos (Figure S2), we used the online program DNAWorks (http://helixweb.nih.gov/dnaworks) developed by Hoover and Lubkowski [25].

**Figure 1:**
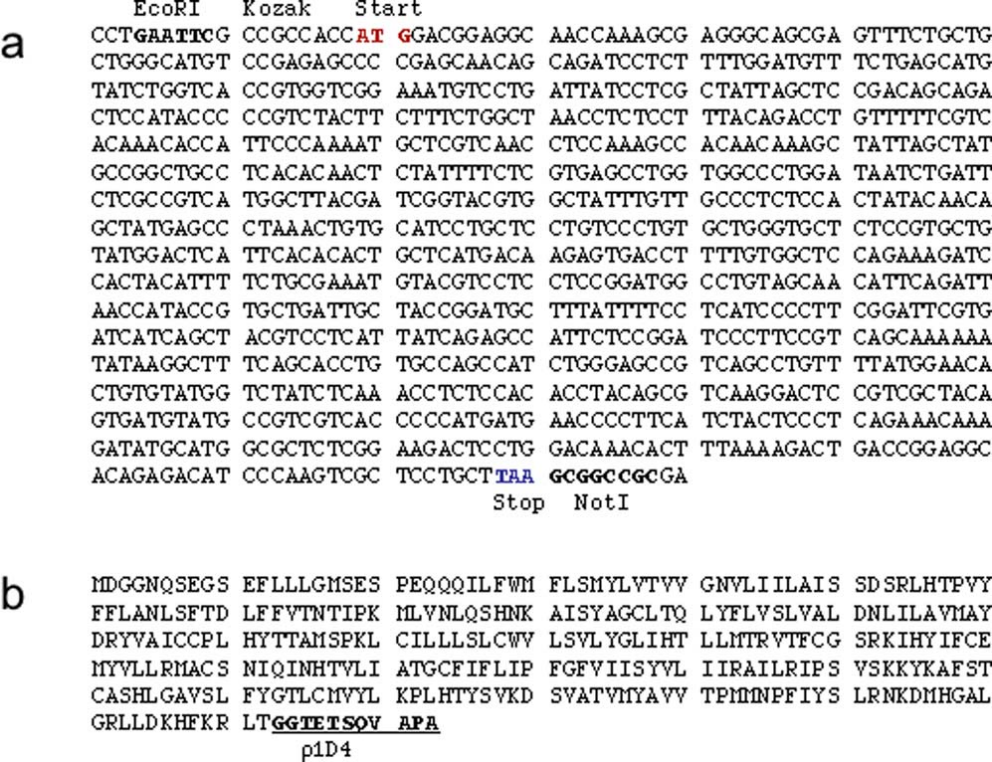
Codon-optimized hOR17-4 sequence. The DNA (A) and corresponding amino acid (B) sequence of synthetic hOR17-4 olfactory receptor gene. The DNA sequence was human codon-optimized and a mammalian Kozak ribosome binding site introduced upstream of the translation start site. Translation start and stop sites as well as restriction cloning sites are indicated. The engineered construct also contains a C-terminal tag (underlined) consisting of a glycine linker followed by a nonapeptide epitope for the monoclonal rho1D4 antibody.

The 312 amino acid sequence of wild-type human olfactory receptor hOR17-4 was obtained from GenBank and directly inputted into the software in protein mode. To adapt the resulting gene for use in our specific expression system and to facilitate purification and detection, the reverse-translated DNA sequence was human codon-optimized and appended with a C-terminal rho1D4 epitope tag (TETSQVAPA) [20]–[22]. The synthetic DNA corresponding to the hOR17-4 gene consisted of 1004 bp, encoding a receptor protein of 323 amino acids (Figure 1). Other than the addition of the rho1D4 tag and linker, the hOR17-4 protein sequence is wild type and completely full length. Noteworthy in the synthesis procedure was the requirement for a high number of PCR cycles (45) during the assembly PCR, presumably due to inefficiencies resulting from non-productive oligonucleotide mispairings.

### Induction of hOR17-4 expression in stable HEK293S cell lines

**Figure 2:**
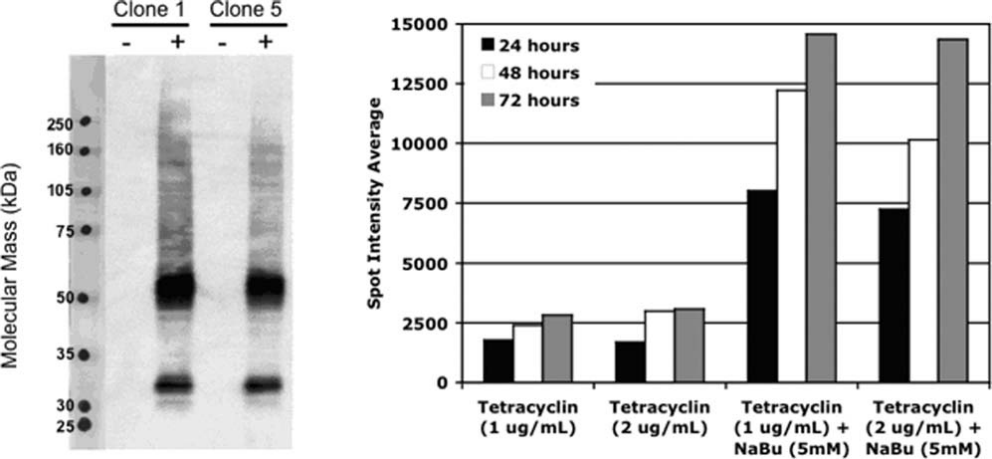
Induction of hOR17-4 in stable HEK293S cell lines. A) Stable inducible cell lines were generated in HEK293S cells using the T-REx system (Invitrogen) expressing hOR17-4 tagged with the rho1D4 tag (TETSQVAPA) at the C-terminus. The pooled cells were then subcloned, expanded, and then tested for induction in media supplemented with (+) or without (−) 1 µg/ml tetracycline for 48 hours. Levels of hOR17-4 were probed via SDS-PAGE western blotting using the rho1D4 antibody. Clones 1 and 5 showed the highest levels of induction while maintaining undetectable background levels in the absence of tetracycline. Clone 5 was selected for all subsequent experiements. B) Addition of sodium butyrate enhances induced expression of hOR17-4. Inducible HEK293 was subjected to a dosage time course using the indicated concentrations of tetracycline and sodium butyrate. Samples were harvested, subjected to dot blot analysis (western blot against rho1D4), and the results quantified by spot densitometry. Tetracycline in conjunction with sodium butyrate increased expression by approximately 4–5 fold over tetracycline alone at all time points tested. Tetracycline was necessary to cause induction, as no expression was detected if sodium butyrate was used alone. Additionally, increasing tetracycline concentration to 2 µg/ml had no significant effect on induction levels.

To minimize the toxic effects of receptor overexpression, stable hOR17-4-inducible HEK293S cell lines were created using the Invitrogen T-REx tetracycline regulation system (Materials and Methods). This allowed large-scale cell culture batches to be grown and then, when desired, concerted production of fresh olfactory receptor to be induced in nearly 100% of the cells. Results for the induction of hOR17-4 expression in two of the subcloned HEK293 lines are shown in Figure 2A. Western immunoblotting using a monoclonal antibody against the rho1D4 tag revealed major immunoreactive bands at approximately 32 kD and 60 kD, which correspond in size to monomeric and dimeric forms of the hOR17-4 receptor. This size pattern has been reported previously for several solubilized olfactory receptors expressed in Sf9 and mammalian cells [26]–[28]. Larger molecular weight complexes were also present, presumably due to aggregation and precipitation of the receptor. As sample boiling only increases the precipitation, it is possible that increased temperatures (above 4°C) caused by the electrophoresis could be causing the receptor to partially aggregate. Thus these high MW species could be a side effect of the SDS-PAGE electrophoresis and not originally present in the solubilized receptor fractions.

The histone deacetlyase inhibitor sodium butyrate has been demonstrated to synergistically enhance expression when used with tetracycline-regulated systems [21]. Induction of hOR17-4 in HEK293S cells using tetracycline in conjunction with sodium butyrate increased expression by approximately 4–5 fold over tetracycline alone at all time points tested (Figure 2B). There was no detectable expression of hOR17-4 in the absence of tetracycline or with sodium butyrate alone. Significant cell toxicity and death was observed in treatments combining tetracycline and sodium butyrate (5 mM) at the 48 and 72 hours time points. Treatment with sodium butyrate or tetracycline alone did not show this toxicity, indicating it to be a result of the high level expression induced by the drugs in conjunction.

**Figure 3:**
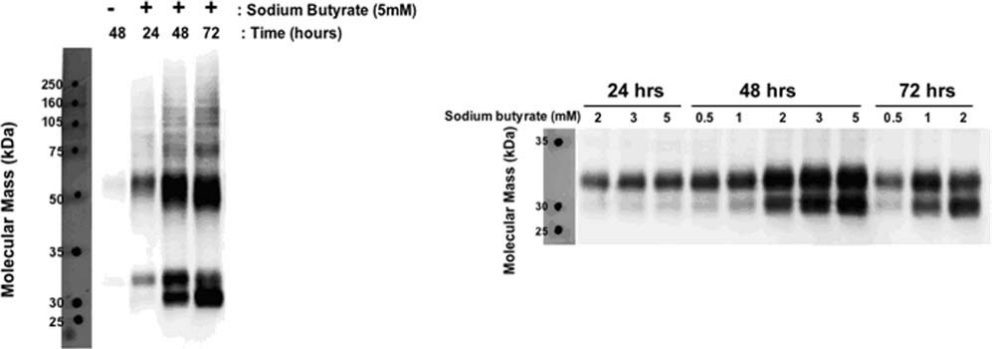
High-level induction correlates with a band shift of hOR17-4. A) Time point samples (from Figure 2) were normalized to total protein content and subjected to SDS-PAGE western blotting against rho1D4. All lanes were treated with 1 µg/ml tetracycline with (+) or without (−) sodium butyrate (5 mM) for the time indicated. B) hOR17-4-inducible HEK293S cells were subjected to a dosage time course using the indicated concentrations of sodium butyrate. All samples were co-treated with 1 µg/ml tetracycline. Samples were normalized to total protein content and subjected to SDS-PAGE western blotting against rho1D4.

To characterize this effect the samples were subjected SDS-PAGE analysis (Figure 3A). The gel showed two monomer band sizes, approximately 30 kD and 32 kD, suggesting distinct monomer forms (and corresponding dimer forms). It is possible these size discrepancies are due to differences in glycosylation of the receptor. Sodium butyrate addition for 24 hours showed a very large increase in expression over tetracycline alone, with the monomer band running at approximately 32 kD. Sodium butyrate addition for longer periods further increased total expression, however this caused the appearance of the additional monomer form running at 30 kD. The 48-hour time point contained roughly equal parts of both forms while the 72-hour time point consisted predominantly of the 30 kD form. To attempt to avoid the aforementioned toxicity we next performed a time course using a range of sodium butyrate concentrations (Figure 3B). We discovered that high levels of expression appeared to correlate with the appearance of the 30 kD form (and correspondingly smaller dimer form) and the observed cytotoxicity noted previously.

For subsequent purification experiments, we selected a treatment consisting of sodium butyrate (1 mM) with tetracycline (1 µg/ml) for 48 hours for attempts to purify primarily the 32 kD form. However, to further compare and characterize both forms, increasing the sodium butyrate concentration to 5 mM could be used, as relatively equal amounts of both monomer forms would be present.

### Purification of heterologously expressed hOR17-4

We incorporated a C-terminal rho1D4-tag utilized by the Khorana lab for rhodopsin purification and detection [20]–[22], namely the nine C-terminal amino acid sequence (TETSQVAPA) against which a specific monoclonal antibody has been generated (rho1D4). This tag has already facilitated early-stage immunoaffinity purifications of several GPCR membrane proteins [29]–[32]. For the initial immunoaffinity purification, we used CNBr-activated Sepharose 4B beads linked to the mouse monoclonal rho1D4 antibody to capture detergent solubilized receptors [21].

**Figure 4:**
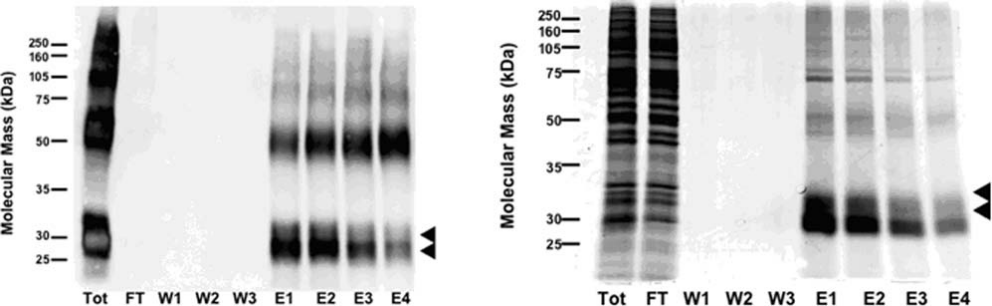
Immunoaffinity purification of hOR17-4. Six 150 mm tissue culture plates of were grown to 90% confluence then induced and treated with tetracycline (1 µg/ml) and sodium butyrate (5 mM) for 48 hours. Plates were scrape harvested, solubilized, and the processed lysate subjected to immunoaffinity purification using rho1D4 antibody linked to sepharose beads for capture. Bound proteins were washed and then eluted using the nonapeptide TETSQVAPA. Samples were subjected to SDS-PAGE followed by either western immunoblotting with rho1D4 antibody (A) or total protein staining with Sypro Ruby (B). Black triangles indicate the 30 and 32 kD monomer forms. Tot, total lysate; FT, flow through; W, wash; E, elution.

We first performed a small-scale purification from six 150 mm culture plates of hOR17-4-inducible HEK293S cells. The plates were treated as to have equal amounts of the 30 kD and 32 kD bands upon harvesting (tetracycline plus 5 mM sodium butyrate, 48 hours after induction). Following a thorough wash procedure to remove non-specific impurities, the bound receptors were eluted by the addition of an excess of epitope peptide (TETSQVAPA). Fractions were subjected to SDS-PAGE followed by either western immunoblotting (Figure 4A) or total protein staining using highly-sensitive SYPRO-Ruby (Figure 4B). The receptor was completely captured by the bead matrix, as no hOR17-4 was detected in the flow through by western blot. The bound OR eluted primarily in the first and second elution fractions. Total yield of hOR17-4 was approximately 30 µg per 150 mm plate. Mass spectrometry analysis on samples isolated from SDS-PAGE gel bands confirmed the identity of putative monomer and dimer protein bands as hOR17-4 receptor (see Materials and Methods).

**Figure 5:**
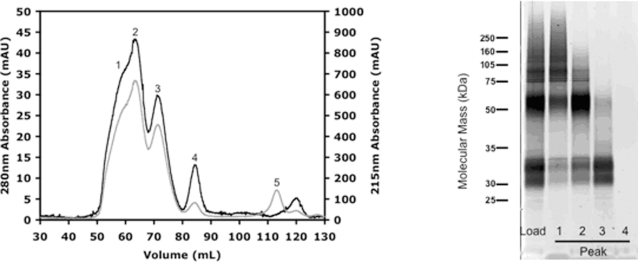
Full purification of hOR17-4 extracted from 50×150 mm culture plates. A) Size exclusion chromatography (SEC) on immunoaffinity-purified hOR17-4. Absorbance was simultaneously recorded at 280 nm (black line, values on left axis) and 215 nm (grey line, values on right axis). The peaks indicated by numbers were pooled and concentrated. Peak 5 consists of the elution nonapeptide from the immunoaffinity purification. B) Total protein staining of SEC peak fractions. Load is the original immunoaffinity purified sample applied to the chromatography column. Peak numbers refer to those designated in (A). Peak 3 contains monomeric hOR17-4 at >90% purity. Total monomer yield was 2.6 µg per 150 mm culture plate.

To expand the system to potential milligram scale, fifty 150 mm culture plates of hOR17-4-inducible HEK293S cells were used. The plates were treated tetracycline plus 5 mM sodium butyrate, 48 hours). The total yield of hOR17-4 following immunoaffinity purification was 1.5 milligrams. To further purify the receptor and to remove the elution peptide, the hOR17-4 was subjected to size exclusion chromatography (SEC) using a gel filtration column on an Äkta HPLC system. Column flowthrough was monitored by UV absorption (280 nm and 215 nm) and separated into fractions by an auto-fraction collector. As seen in Figure 5A, five distinct peaks were observed. The peak fractions were then pooled, concentrated and subjected to SDS-PAGE followed by total protein staining. As seen in Figure 5B, peak 3 contains monomeric hOR17-4 (>90% purity) while earlier peaks contained largely dimeric (peak 2) and aggregated/oligomerized (peak 1) forms. Peak 4 showed no visible protein and peak 5 corresponds to the residual elution peptide (TETSQVAPA) from the immunoaffinity purification. The final yield of purified hOR17-4 monomer was 0.13 milligrams (2.6 µg per plate). Thus, using a two-step procedure we have successfully obtained significant amounts of hOR17-4 in a highly pure form.

## Discussion

In this study, we successfully developed methods for the construction of inducible mammalian cell lines that generate large quantities of olfactory receptor on demand. To our knowledge this is the first olfactory receptor to be purified from a mammalian cell line. Currently, we have demonstrated the production of the human olfactory receptor hOR17-4 in a stable tetracycline-inducible human embryonic kidney cell line (HEK293S). Expressed OR genes were fabricated from scratch using PCR-based gene synthesis (polymerase construction and amplification, aka PCA), which facilitated codon optimization for high level expression and attachment of affinity tags for detection and purification. The HEK293S cells can be grown and OR expression induced in adherent cultures (yield of ∼30 micrograms/150-mm plate). Using methods originally adapted from the production and purification of the bovine GPCR rhodopsin [20]–[22] and further optimized (including the full-spectrum screening of over 70 detergents), the olfactory receptor is solubilized and extracted from the cells using fos-choline-14. The OR protein is then isolated using a two-step purification method (immunoaffinity followed by size-exclusion chromatography) which yields hOR17-4 monomer at greater than 90% purity (Figure 5B).

There have been a host of previous studies that have expressed and studied olfactory receptors in native and heterologous systems. However, to date there has not been a case where olfactory receptors have been overexpressed and purified to homogeneity in significant quantities. While purification of ORs has been attempted in bacterial [33] and Sf9 insect systems [26]–[27], these were unable to produce large quantities of native full-length olfactory receptor. Additionally, these organisms lack mammalian post-translational machinery and thus purified receptors may be improperly folded or missing critical modifications. It is known that most, if not all, GPCRs are glycosylated, and indeed the olfactory receptors have conserved N-linked glycosylation sites (Asn-X-Ser/Thr) at their N-terminus [34]–[35]. Several studies have indicated that loss of glycosylation can lead to improper folding and targeting, resulting in decreased function and compromised structure [36]–[37]. Loss of either N-terminal glycosylation site (Asn-2 or Asn-15) of rhodopsin is sufficient to cause loss of signal transduction despite no apparent change in localization or folding [38]–[39]. This indicates that purification of olfactory receptors from mammalian systems might be crucial for functional expression and purification.

### Characterization of purified hOR17-4

It is interesting to note that the engineered hOR17-4-rho protein (with theoretical molecular mass of 36.2 kD) migrates slightly faster than expected on SDS-PAGE gels (30 kD and 32 kD). One possible explanation for this discrepancy is incomplete receptor denaturation by SDS since boiling the samples results in aggregation. Indeed, many other membrane proteins have been found to migrate faster than their actual size on SDS-PAGE, and this has been reported for other olfactory receptors as well [27], [33]. We believe both monomer forms of the expressed hOR17-4 receptor to be intact, full-length proteins as evidenced by: 1) detection by the C-terminal rho1D4 monoclonal antibody, 2) detection by custom anti-hOR17-4 polyclonal antibodies raised against the hOR17-4 N-terminus or C-terminus, 3) detection of a 30 kD N-terminally 6xHis-tagged hOR17-4 variant using anti-His antibodies (data not shown). Additionally, we see only the 32 kD form at low levels of induction while the 30 kD form only begins to appear at higher levels of induction. One explanation is that the receptor is glycosylated at low induction but that the toxicity associated with higher expression causes the receptor to begin to accumulate in the ER and not be properly processed.

The appearance of two distinct hOR17-4 monomer bands following purification could pose a problem for structural studies using X-ray crystallography, since typically a high degree of protein homogeneity is required for protein crystallization. We initially believed that it was possible to obtain primarily 32 kD form using lower levels of induction (Figure 3B). However, we have observed that the rho1D4 immunoaffinity purification appears to increase the proportion of 30 kD monomer form relative to 32 kD form given the original treatment. For example, the treatment dosages for the small-scale (Figure 4) and large-scale (Figure 5) purifications were chosen (based on data in Figure 3B) to result in equal amounts of both forms or primarily 32 kD form, respectively. However, following immunoaffinity purification the resulting elution samples were enriched in the 30 kD monomer form. One hypothesis is that the 30 kD (potentially non-glycosylated form) binds more readily to the rho1D4-coupled bead matrix. Therefore, to obtain truly homogeneous hOR17-4 monomer it might be necessary to use even lower levels of induction where no 30 kD form exists. Another option would be to mutate the asparagine in the consensus N-glycosylation site of the receptor, located at amino acid position 5. We predict such a change to result in exclusive production of the 30 kD monomer form. However, the functional effect of abolishing glycosylation on the receptor is unknown.

There have been numerous reports in the literature citing difficulties in expressing functional olfactory receptors in heterologous systems. The main problem seems to be improper membrane targeting and resulting cytosolic localization of the majority of ORs [35]–[36], [40]–[41]. Several studies have been able to alleviate this problem by the addition of putative membrane import signals to the N-terminus of the olfactory receptor [42]–[43]. However, more recent studies [44]–[45] have expressed functional olfactory receptors in HEK293 cells without the use of such signal tags. We also investigated the use of an N-terminal “membrane import” sequence composed of the first twenty amino acids of bovine rhodopsin but found no significant increase in receptor yield or localization (results not shown).

Our largest adherent culture experiment consisted of fifty 150 mm tissue culture plates. While the total yield of crude hOR17-4 following immunoaffinity purification was 1.5 milligrams, the subsequent size exclusion chromatography and associated concentration steps reduced this yield to 0.13 milligrams of purified hOR17-4 monomer (2.6 µg per plate). The monomer was >90% pure, with the only other major contaminating band consisting of hOR17-4 dimer. While this represents a significant milestone, using adherent culture for milligram-scale purification of the receptor monomer poses a substantial challenge. However, the HEK293S cell line is capable of suspension culture, and we plan to scale-up purification yields by adapting the system to culture in a large volume (5–10 liter) liquid bioreactor. The high cell densities allowed should allow the production and subsequent purification of milligram quantities of olfactory receptors, which will be necessary for future experiments in determining receptor structure and function.

### General application for the purification of other olfactory receptors

Here we show that a GPCR olfactory receptor gene can be designed, synthesized, placed into an inducible mammalian expression system and the resulting full-length protein purified to near homogeneity in a two-step process. In addition to our experiments on hOR17-4, we have constructed optimized synthetic genes for several mouse olfactory receptors and initial expression trials have proven successful. The small size of the rho1D4 tag and its extremely mild elution conditions provide a minimum of disruption to the purified protein. In contrast to previous attempts at OR purification using bacterial systems which required fusion to GST and truncated or mutated OR protein sequences [33], the system described here allows for the production of full-length wild-type OR. The application of this technique to other olfactory receptors could feasibly lead to a generalized method for obtaining large quantities of any olfactory receptor in a rapid and simple manner. Such methods could prove extremely useful in elucidating the structural and functional mechanism(s) of olfactory receptors and in their integration into OR-based bionic sensing devices.

### Simple and adaptable methods

Membrane proteins are natural nanobiodevices; they perform exquisitely fine functions, from specific ion channels, chemical transporters, signal transduction, and information processing, to photosynthesis for direct solar energy harvesting. In order to accelerate determination of membrane protein structural studies, simple and reliable methods are necessary to attract more and more researchers to study these elusive membrane proteins and use them to fabricate new bionic devices. We here show that the GPCR olfactory receptor gene can be bioengineered, placed into an inducible system and to purify to near homogeneity in a straightforward 2-step process. Our effort to purify hOR17-4 is a very important step not only for us to understand the secret of smell, but also to design new bionic nose sensing devices.

## Materials and Methods

### Gene Construction

The protein sequence for hOR17-4 (also known as OR1D2) was obtained from GenBank (NCBI Accession # NP002539). DNAWorks online software (http://helixweb.nih.gov/dnaworks) was used in protein mode to design the synthetic gene and parse it into an oligonucleotide set. To adapt the synthetic hOR17-4 olfactory receptor gene for use in mammalian cell expression and purification, the following sequence modifications were made: i) human codon optimization; ii) addition of a C-terminal rho1D4 epitope tag (TETSQVAPA) preceded by a two glycine linker to facilitate detection and purification; iii) addition of a Kozak consensus sequence (GCCACCACC) immediately 5′ to the ATG start codon; iv) addition of an EcoRI restriction site at the 5′ end and a NotI restriction site at 3′ end of the gene to enable cloning into expression vectors. The synthetic hOR17-4 gene consisted of 1004 bp, of which 969 bp code for the 323 amino acid hOR17-4-GlyGly-rho1D4 protein. The designed oligonucleotide primers were purchased from IDT (Coralville, IA) with a maximum length of 45 bp (Figure S2). PCR-based gene synthesis was performed using a 2-step assembly/amplification protocol (Figure S1) [46] with the exception that the assembly PCR was run for 45 cycles. PCR reactions were then analyzed by gel electrophoresis and stained with ethidium bromide. Full-length product was excised, extracted, and then digested with the pertinent restriction enzymes. The genes were then ligated into the T-REx pcDNA4/To inducible expression plasmid (Invitrogen, Carlsbad, CA), sequenced, and a correct clones grown up using a MaxiPrep kit (Qiagen, Valencia, CA). The plasmid containing the optimized hOR17-4 gene was designated pcDNA4/To-hOR17-4-rho1D4.

### Generation of Stable Inducible Cell Lines

HEK293S (suspension adapted HEK293 cells) containing the stable expression of pcDNA6/Tr (Invitrogen) which encodes the Tet repressor protein (TetR) had previously been generated and cloned [21]. HEK293S cell monolayers were grown in DMEM/F12 with GlutaMAX (Invitrogen catalog # 10565-042) supplemented with fetal bovine serum (10%), HEPES (15 mM), non-essential amino acids (0.1 mM), sodium pyruvate (0.5 mM), penicillin (100 units/ml), streptomycin (100 µg/ml) and grown at 37°C at 5% CO_2_. All tissue culture and media components were purchased from Invitrogen unless otherwise noted. The pcDNA4/To-hOR17-4-rho1D4 plasmid was then transfected into these cells using Lipofectamine 2000 and after 48 hours cells were subjected to drug selection in 5 µg/ml blasticidin and 250 µg/ml zeocin for 2 weeks and then subcloned. 28 colonies were expanded and screened for inducible expression using media supplemented with or without 1 µg/ml tetracycline for 48 hours. Samples were then scrape harvested, solubilized in phosphate buffered saline (PBS) with 2% w/v Fos-Choline-14 (Anatrace, Maumee, OH) and Complete Protease Inhibitor Cocktail (Roche, Basel, CH) for 1 hour at 4°C. Expression was assayed via dot blotting and SDS-PAGE western blotting using the mouse monoclonal antibody rho1D4. Clone 5, the colony showing the best expression of hOR17-4 under induction conditions while maintaining undetectable expression without induction, was selected and expanded into large-scale culture and used for all subsequent experiments. The hOR17-4-inducible HEK293S cell line was maintained using 5 µg/ml blasticidin and 250 µg/ml zeocin.

### Cell Extract Preparation

Buffers used were as follows: PBS buffer: 137 mM NaCl, 2.7 mM KCl, 1.8 mM KH_2_PO_4_, 10 mM Na_2_HPO_4_ (pH 7.4); Solubilization buffer: PBS containing Complete Protease Inhibitor Cocktail (Roche, Basel, Switzerland) and 2% wt/vol FC14; and Wash buffer: PBS containing 0.2% FC14; Elution buffer: Wash buffer containing 100 µM Ac-TETSQVAPA-CONH_2_ elution peptide. The detergent FC14 was purchased from Anatrace (Maumee, OH). Sodium butyrate was purchased from Sigma (Saint Louis, MO).

For initial dosage and time course experiments, hOR17-4-inducible HEK293S cells were grown to 80–90% confluency at 37°C in 6-well tissue culture plates, treated as indicated and then scrape harvested into ice-cold PBS containing Complete Protease Inhibitor Cocktail. The hOR17-4 was then solubilized by resuspending the cell pellets in 150 µl solubilization buffer and rotating for 1 hour at 4°C. The non-solubilized fraction was then pelleted using at 13,000 g for 30 minutes. The supernatant was then removed and analyzed by SDS-PAGE.

For purification experiments, up to fifty 150 mm tissue culture plates were used per experiment. Briefly, hOR17-4-inducible HEK293S cells were seeded at a density of 5×10^6^ cells per 150 mm dish and grown for 72 hours at 37°C, at which point they reached 80–90% confluency. The cells were then induced with medium containing tetracycline (1 µg/ml) plus sodium butyrate (as indicated). After 48 hours, the cells were harvested by scraping (at 4°C) each plate into 2 ml PBS containing Complete Protease Inhibitor Cocktail. The cells were then pooled and snap frozen in liquid nitrogen and stored at −80°C until purification was carried out. On the day of purification, cells were thawed on wet ice and spun down by centrifugation at 4000 g for 1 minute. All further steps were performed at 4°C unless noted. The hOR17-4 was then solubilized by resuspending the cells in solubilization buffer (1–2 ml per 150 mm plate) and rotating for 4 hours. The non-solubilized fraction was then pelleted using an ultracentrifuge at >100,000 g for 30 minutes. The resulting supernatant was removed and put at 4°C. A small amount of supernatant (100 µl) was set aside, labeled “total lysate” and stored at −20°C. The remainder was directly applied to immunoaffinity purification.

### Immunoblotting and Total Protein Staining

Samples were assayed via polyacrylamide gel electrophoresis (SDS-PAGE) under both reducing and denaturing conditions. Samples were prepared and loaded according to standard Novex gel protocols with the exception that the samples were incubated at room temperature prior to loading, as boiling caused membrane protein aggregation. Full Range Rainbow (GE Healthcare, Waukesha, WI) molecular weight marker was loaded as the protein size standard. Samples were resolved on Novex 10% Bis-Tris SDS-PAGE gels (Invitrogen) were run using NuPAGE MOPS buffer at 100 V and were subsequently transferred to a 0.45 µm nitrocellulose membrane and subjected to western immunoblotting using the rho1D4 as primary antibody, followed by a secondary HRP-linked goat anti-mouse IgG (Pierce, Rockford, IL) and detection using the ECL-Plus Kit (GE Healthcare). For total protein staining, SDS-PAGE gels were run as above, stained using SYPRO-Ruby (a more sensitive alternative to Coomassie; Invitrogen), and visualized by fluorescence using UV transillumination (excitation wavelength 300 nm). All western blot and SYPRO-Ruby images were captured using a Fluor Chem gel documentation system (Alpha Innotech, San Leandro, CA).

### Immunoaffinity Purification

For immunoaffinity purification we utilized rho1D4 monoclonal antibody (Cell Essentials, Cambridge, MA) chemically linked to CNBr-activated Sepharose 4B beads (GE Healthcare). The rho1D4 elution peptide Ac-TETSQVAPA-CONH_2_ was synthesized by CBC Scientific (San Jose, CA). Rho1D4-sepharose immunoaffinity purification has been described previously [21]. Briefly, the cell extract supernatant was mixed with rho1D4-coupled sepharose bead slurry (binding capacity 0.7 mg/ml) and rotated overnight at 4°C to capture the rho1D4-tagged olfactory receptors. The beads were then pelleted by centrifugation at 2000 g for one minute and the supernatant collected, labeled as “flow-through” and saved for future analysis. The beads were then re-suspended in 100 bead volumes of cold wash buffer (PBS+0.2% Fos-Choline-14), rotated for 10 minutes at 4°C, then re-pelleted. A total of five washes were carried out and 100 µl of each sequential wash was saved for subsequent analysis. After the final wash, the beads were pelleted again and transferred to a new tube for elution. A series of five elutions (each rotated 1 hour at room temperature) was then carried out, each using 1 bead volume of elution buffer (PBS+0.2% FC14+100 µM TETSQVAPA peptide). Total protein concentration was measured using BCA assay (Pierce).

### Mass Spectrometry

Immunoaffinity-purified samples of hOR17-4 were separated via SDS-PAGE, stained with SYPRO-Ruby and gel bands at 30 kD, 32 kD, and 60 kD were excised into sterile, methanol-rinsed microcentrifuge tubes. The samples subjected to trypsin digestion and the resulting fragments analyzed by Ion Trap LCMS for protein identification by the MIT Biopolymers Laboratory (Cambridge, MA). All bands were identified as hOR17-4, indicating monomeric and dimeric forms.

### Size Exclusion Chromatography

For further purification, hOR17-4 proteins were subjected to gel filtration chromatography using a HiLoad 16/60 Superdex 200 column on an Äkta Purifier HPLC system (GE Healthcare). The column was first equilibrated using wash buffer (PBS+0.2% w/v Fos-Choline-14). Pooled hOR17-4 elution fractions from the rho1D4 immunoaffinity purification were concentrated to 0.75 mg/ml using a 10 kD MWCO filter column (Millipore, Billerica, MA) and then applied to the Äkta system. After loading, the column was run with wash buffer at 1 ml/min and column flowthrough monitored via UV absorbance at 280 nm and 215 nm. Protein fractions were collected using an automated fraction collector. Peak fractions were then pooled, concentrated and subjected to SDS-PAGE and analysis via Sypro Ruby staining. Total protein concentration was measured using BCA assay (Pierce).

## Supporting Information

Figure S1 PCR-based Gene Synthesis. Fabrication of the custom hOR17-4-rho gene was accomplished using a two-step PCR method using an overlapping oligonucleotide set designed using DNAWorks. (A) The first step is an assembly PCR in which all oligos function as both primer and template to effectively extend their 3′ ends with successive PCR cycles. After many cycles of assembly, the full-length gene is constructed. However, the assembly reaction contains not only full-length product but also a mixture of all other extended oligos. Thus a second PCR reaction (B) is performed using a small amount of the assembly PCR reaction as template and the two terminal oligos (oligos A and L) as primers in order to selectively amplify the full-length gene product.
(1.17 MB TIF)

Figure S2 Primers used for the PCR-based synthesis of the engineered hOR17-4 gene. A total of 50 oligonucleotides were used to construct the synthetic gene (25 sense strand oligos, labeled S1-25) and 25 anti-sense strand oligos, labeled AS1-25).
(0.04 MB DOC)
