# Supplementary figures and images for: Study of a Synthetic Human Olfactory Receptor 17-4: Expression and Purification from an Inducible Mammalian Cell Line
Source: PLoS One. 2008 Aug 6;3(8):e2920. doi: 10.1371/journal.pone.0002920 (PMC2488374; doi:10.1371/journal.pone.0002920)

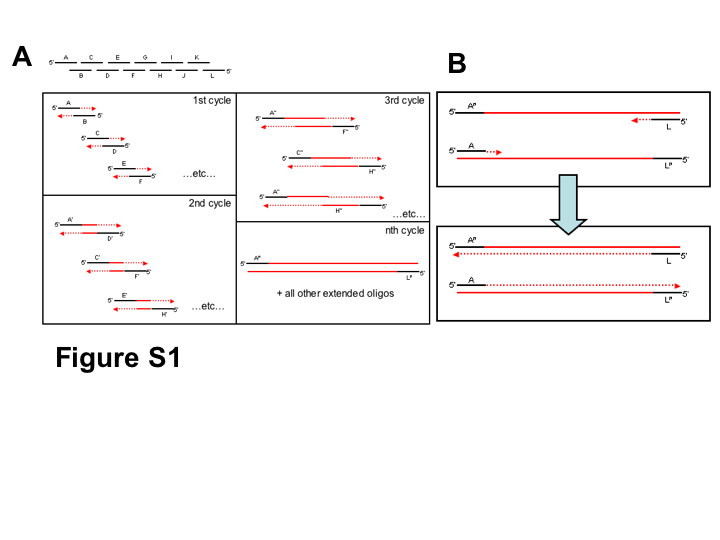

Supplement: Figure S1 — PCR-based Gene Synthesis. Fabrication of the custom hOR17-4-rho gene was accomplished using a two-step PCR method using an overlapping oligonucleotide set designed using DNAWorks. (A) The first step is an assembly PCR in which all oligos function as both primer and template to effectively extend their 3′ ends with successive PCR cycles. After many cycles of assembly, the full-length gene is constructed. However, the assembly reaction contains not only full-length product but also a mixture of all other extended oligos. Thus a second PCR reaction (B) is performed using a small amount of the assembly PCR reaction as template and the two terminal oligos (oligos A and L) as primers in order to selectively amplify the full-length gene product. (1.17 MB TIF) [file pone.0002920.s001.tif]
