# Supplementary material for: Study of a Synthetic Human Olfactory Receptor 17-4: Expression and Purification from an Inducible Mammalian Cell Line
Source: PLoS One. 2008 Aug 6;3(8):e2920. doi: 10.1371/journal.pone.0002920 (PMC2488374; doi:10.1371/journal.pone.0002920)
Supplement: Figure S2 — Primers used for the PCR-based synthesis of the engineered hOR17-4 gene. A total of 50 oligonucleotides were used to construct the synthetic gene (25 sense strand oligos, labeled S1-25) and 25 anti-sense strand oligos, labeled AS1-25). (0.04 MB DOC) [file pone.0002920.s002.doc]

Figure S2. Primers used for the PCR-based synthesis of the engineered hOR17-4 gene. A total of 50 oligonucleotides were used to construct the synthetic gene (25 sense strand oligos, labeled S1-25) and 25 anti-sense strand oligos, labeled AS1-25).

**Primer** **Sequence** **# bp**

S1: CCTGAATTCGCCGCCACCATGGACGGAGGCAA 32

S2: CCAAAGCGAGGGCAGCGAGTTTCTGCTGCTGG 32

S3: GCATGTCCGAGAGCCCCGAGCAACAGCAGATCCT 34

S4: CTTTTGGATGTTTCTGAGCATGTATCTGGTCACCGTGGTCG 41

S5: GAAATGTCCTGATTATCCTCGCTATTAGCTCCGACAGCAGACTC 44

S6: CATACCCCCGTCTACTTCTTTCTGGCTAACCTCTCCTTTACAGA 44

S7: CCTGTTTTTCGTCACAAACACCATTCCCAAAATGCTCGTCAA 42

S8: CCTCCAAAGCCACAACAAAGCTATTAGCTATGCCGGCTG 39

S9: CCTCACACAACTCTATTTTCTCGTGAGCCTGGTGGCCC 38

S10: TGGATAATCTGATTCTCGCCGTCATGGCTTACGATCGGTAC 41

S11: GTGGCTATTTGTTGCCCTCTCCACTATACAACAGCTATGAGCC 43

S12: CTAAACTGTGCATCCTGCTCCTGTCCCTGTGCTGGG 36

S13: TGCTCTCCGTGCTGTATGGACTCATTCACACACTGCTCA 39

S14: TGACAAGAGTGACCTTTTGTGGCTCCAGAAAGATCCACTACATTT 45

S15: TCTGCGAAATGTACGTCCTCCTCCGGATGGCCTGTAG 37

S16: CAACATTCAGATTAACCATACCGTGCTGATTGCTACCGGATGCTT 45

S17: TATTTTCCTCATCCCCTTCGGATTCGTGATCATCAGCTACGTC 43

S18: CTCATTATCAGAGCCATTCTCCGGATCCCTTCCGTCAGCAAAA 43

S19: AATATAAGGCTTTCAGCACCTGTGCCAGCCATCTGGGAG 39

S20: CCGTCAGCCTGTTTTATGGAACACTGTGTATGGTCTATCTCAAAC 45

S21: CTCTCCACACCTACAGCGTCAAGGACTCCGTCGCTA 36

S22: CAGTGATGTATGCCGTCGTCACCCCCATGATGAACCC 37

S23: CTTCATCTACTCCCTCAGAAACAAAGATATGCATGGCGCTCTC 43

S24: GGAAGACTCCTGGACAAACACTTTAAAAGACTGACCGGAGG 41

S25: CACAGAGACATCCCAAGTCGCTCCTGCTTAAGCGGC 36

AS1: CTTCTCGCGGCCGCTTAAGCAGGAG 25

AS2: CGACTTGGGATGTCTCTGTGCCTCCGGTCAGTCTTTTAAAG 41

AS3: TGTTTGTCCAGGAGTCTTCCGAGAGCGCCATGCATATC 38

AS4: TTTGTTTCTGAGGGAGTAGATGAAGGGGTTCATCATGGGGGTG 43

AS5: ACGACGGCATACATCACTGTAGCGACGGAGTCCTTGA 37

AS6: CGCTGTAGGTGTGGAGAGGTTTGAGATAGACCATACACAGTGT 43

AS7: TCCATAAAACAGGCTGACGGCTCCCAGATGGCTGGC 36

AS8: ACAGGTGCTGAAAGCCTTATATTTTTTGCTGACGGAAGGGATC 43

AS9: CGGAGAATGGCTCTGATAATGAGGACGTAGCTGATGATCACGAA 44

AS10: TCCGAAGGGGATGAGGAAAATAAAGCATCCGGTAGCAATCAG 42

AS11: CACGGTATGGTTAATCTGAATGTTGCTACAGGCCATCCGGAG 42

AS12: GAGGACGTACATTTCGCAGAAAATGTAGTGGATCTTTCTGGAGC 44

AS13: CACAAAAGGTCACTCTTGTCATGAGCAGTGTGTGAATGAGTC 42

AS14: CATACAGCACGGAGAGCACCCAGCACAGGGACAG 34

AS15: GAGCAGGATGCACAGTTTAGGGCTCATAGCTGTTGTATAGTGG 43

AS16: AGAGGGCAACAAATAGCCACGTACCGATCGTAAGCCATGA 40

AS17: CGGCGAGAATCAGATTATCCAGGGCCACCAGGCTC 35

AS18: ACGAGAAAATAGAGTTGTGTGAGGCAGCCGGCATAGCTAATAG 43

AS19: CTTTGTTGTGGCTTTGGAGGTTGACGAGCATTTTGGGAATG 41

AS20: GTGTTTGTGACGAAAAACAGGTCTGTAAAGGAGAGGTTAGCCA 43

AS21: GAAAGAAGTAGACGGGGGTATGGAGTCTGCTGTCGGAGC 39

AS22: TAATAGCGAGGATAATCAGGACATTTCCGACCACGGTGACCA 42

AS23: GATACATGCTCAGAAACATCCAAAAGAGGATCTGCTGTTGCTCG 44

AS24: GGGCTCTCGGACATGCCCAGCAGCAGAAACTCG 33

AS25: CTGCCCTCGCTTTGGTTGCCTCCGTCCATGG 31
